# COSMIC: the Catalogue Of Somatic Mutations In Cancer

**DOI:** 10.1093/nar/gky1015

**Published:** 2018-10-29

**Authors:** John G Tate, Sally Bamford, Harry C Jubb, Zbyslaw Sondka, David M Beare, Nidhi Bindal, Harry Boutselakis, Charlotte G Cole, Celestino Creatore, Elisabeth Dawson, Peter Fish, Bhavana Harsha, Charlie Hathaway, Steve C Jupe, Chai Yin Kok, Kate Noble, Laura Ponting, Christopher C Ramshaw, Claire E Rye, Helen E Speedy, Ray Stefancsik, Sam L Thompson, Shicai Wang, Sari Ward, Peter J Campbell, Simon A Forbes

**Affiliations:** 1 Wellcome Sanger Institute, Wellcome Genome Campus, Hinxton, Cambridge CB10 1SA, UK; 2 Astex Pharmaceuticals, 436 Cambridge Science Park, Cambridge CB4 0QA, UK; 3 Open Targets, Wellcome Genome Campus, Hinxton, Cambridge CB10 1SD, UK

## Abstract

COSMIC, the Catalogue Of Somatic Mutations In Cancer (https://cancer.sanger.ac.uk) is the most detailed and comprehensive resource for exploring the effect of somatic mutations in human cancer. The latest release, COSMIC v86 (August 2018), includes almost 6 million coding mutations across 1.4 million tumour samples, curated from over 26 000 publications. In addition to coding mutations, COSMIC covers all the genetic mechanisms by which somatic mutations promote cancer, including non-coding mutations, gene fusions, copy-number variants and drug-resistance mutations. COSMIC is primarily hand-curated, ensuring quality, accuracy and descriptive data capture. Building on our manual curation processes, we are introducing new initiatives that allow us to prioritize key genes and diseases, and to react more quickly and comprehensively to new findings in the literature. Alongside improvements to the public website and data-download systems, new functionality in COSMIC-3D allows exploration of mutations within three-dimensional protein structures, their protein structural and functional impacts, and implications for druggability. In parallel with COSMIC’s deep and broad variant coverage, the Cancer Gene Census (CGC) describes a curated catalogue of genes driving every form of human cancer. Currently describing 719 genes, the CGC has recently introduced functional descriptions of how each gene drives disease, summarized into the 10 cancer Hallmarks.

## INTRODUCTION

COSMIC, the Catalogue Of Somatic Mutations In Cancer, draws together the available information about the effects of somatic mutations across the range of human cancers. As described previously (1), the primary data in COSMIC are derived directly from the scientific literature by expert manual curators, who read and digest journal articles and extract the detailed mutation data within, along with any additional information such as environmental factors or patient pre-disposition that may be accessible. In parallel with this broad-ranging manual curation process, a second curation track brings into COSMIC a wealth of larger-scale but more narrowly-focussed data from systematic screens, via the major cancer data portals and from the supplementary tables and downloadable files associated with curated papers. Data from these two curation strands combine to give COSMIC an unrivalled breadth and depth of coverage, making it the primary resource for the exploration of the aetiology and landscape of mutations in human cancer.

**Figure 1.**
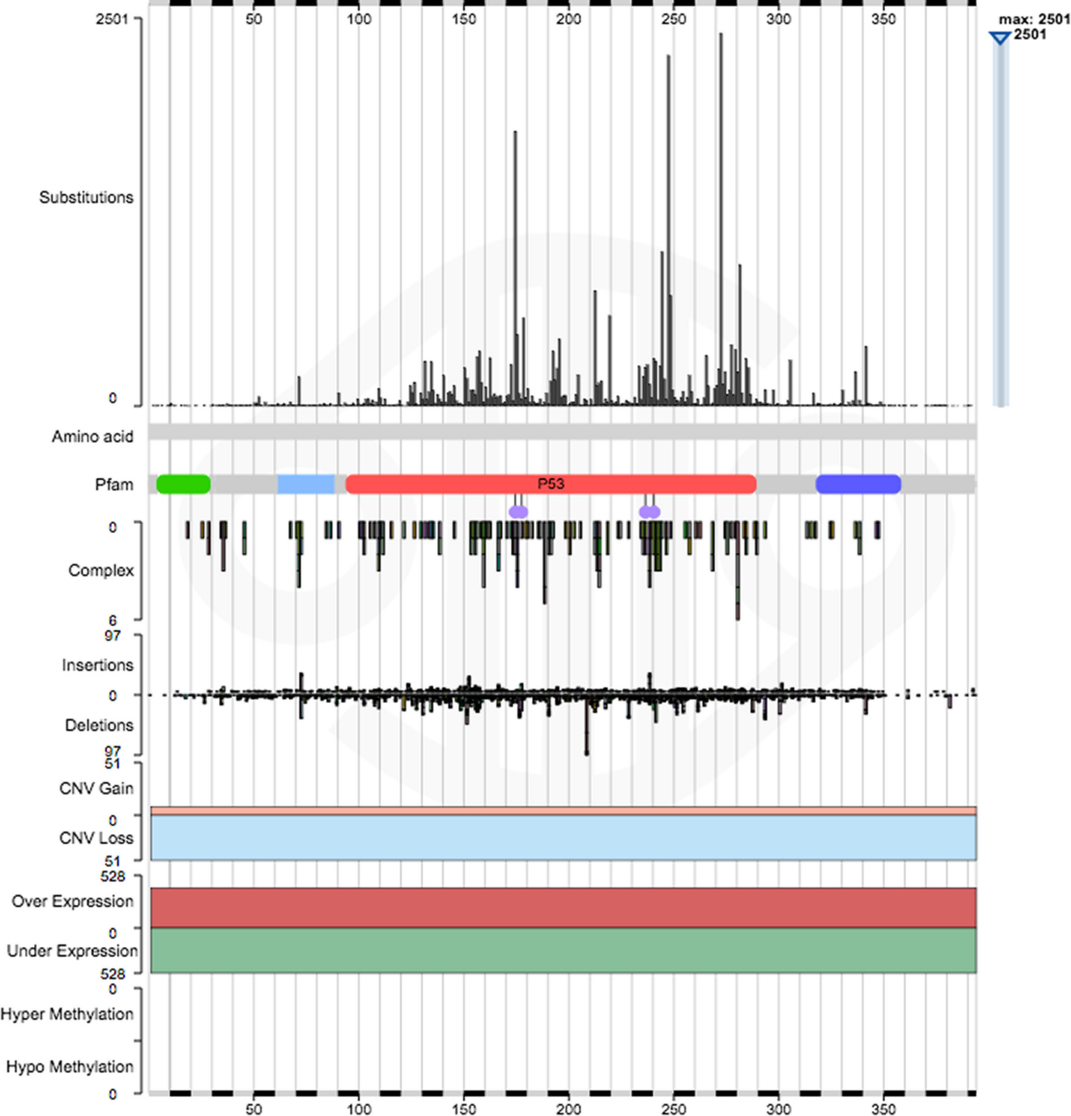
The gene histogram for the tumour suppressor gene TP53, showing the range of data available for TP53. When showing the amino acid sequence, as here, the top panel displays the single residue mutations across the gene. When zoomed in sufficiently, the amino acid sequence itself is shown beneath. If the gene has Pfam families associated with it, these are shown next, along with any Pfam annotations, such as the metal ion binding sites as here in TP53. Next, we show the presence of complex mutations and insertions/deletions, followed by copy number gain/loss, expression levels and methylation. A toggle in the filter panel in the gene page may be used to switch the view from amino acid to nucleic acid coordinate systems, and the switch is reflected in the exact data that is available in the histogram.

Growing from an initial survey of only four genes in 2004 (2), COSMIC today encompasses every human gene, describing 5 977 977 coding mutations across 1 391 372 samples. A total of 223 key cancer genes are subject to deep, exhaustive curation by expert scientists, gathering information from 26 251 papers to date. This is merged with genome-wide annotations from 466 whole genome and large-scale systematic screens publications, as well as open-access data from The Cancer Genome Atlas (TCGA) (3) and the International Cancer Genome Consortium (ICGC) (4). Data within COSMIC are updated constantly and released on a regular, three-monthly cycle, guaranteeing four releases per year. Table 1 shows a summary of the data in the most recent release, v86 (August 2018). Data are presented in a powerful and comprehensive website (https://cancer.sanger.ac.uk/cosmic), which collects and presents the wealth of data in tabular form or as interactive visualizations, such as the gene histogram (Figure 1).

**Table 1.** Total contents in version 86 of the COSMIC database (August 2018)

**1 391 372**	Tumour samples
**5 977 977**	Coding Mutations
**26 251**	Manually Curated Publications
**19 368**	Gene Fusions
**35 480**	Whole Genomes/Exomes across **457** studies/papers
**1 179 545**	Copy Number Variants
**9 147 833**	Gene Expression Variants
**7 879 142**	Differentially Methylated CpGs
**19 721 019**	Non-coding Variants

The main COSMIC resource is complemented by additional datasets and tools. The COSMIC Cell Lines Project (https://cancer.sanger.ac.uk/cell_lines) comprises data from the full exome sequencing and molecular profiling of 1015 cell lines at the Wellcome Sanger Institute, and aims to systematically characterize the genetics and genomics of large numbers of cancer cell lines. The Cancer Gene Census (CGC) (https://cancer.sanger.ac.uk/census) (5) identifies and describes every gene that has a demonstrable role across all forms of human cancer. COSMIC-3D is a new tool linking the detailed sequence-level mutation data in COSMIC with the rich protein-structural data in the Protein Data Bank, facilitating structure, function, and druggability analysis.

## COSMIC CONTENTS

### Cancer gene census

The CGC (https://cancer.sanger.ac.uk/census) is an ongoing, long-term project within COSMIC to catalogue all genes that are causally implicated in cancer through somatic and germline mutations. Based on an original collection of 291 cancer genes (5), the latest Census (COSMIC v86, August 2018) describes 719 genes (6), including their contribution to disease causation, the types of mutations causing dysfunction of the gene in cancer, and the types of cancer in which mutations have been observed with increased frequency.

The evaluation process for CGC candidate genes starts with a search for the presence of somatic mutation patterns typical for cancer genes. Having identified a candidate gene, a thorough literature review is performed to identify the biological functions of the gene and to establish how mutations cause dysfunction of that gene to promote oncogenic transformation. At this stage the gene can be classified as an oncogene, a tumour suppressor gene (TSG), or both. If described in the context of oncogenic fusions, such a gene is classified as a ‘fusion gene’.

After a recent major expansion, the CGC comprises two ‘tiers’, into which genes are classified depending on strength of evidence supporting their cancer-promoting role. Tier 1 genes are characterized by the presence of mutational patterns that strongly support their involvement in cancer aetiology, along with evidence of how the gene's dysfunction impacts the hallmarks of cancer (7). Qualification for Tier 1 requires at least two publications from two independent groups, which describe somatic mutations in the gene in at least one type of cancer. Additionally, at least two independent publications must provide evidence of functional involvement of the gene in biological processes driving cancer. The details of the curation process and the criteria for gene qualification to the CGC have been described previously (6).

Tier 2 of the CGC encompasses genes with extensive literature evidence for their participation in tumour development but which have less robust evidence supporting mutational patterns or functional consequence. The evidence is assessed independently by at least two postdoctoral scientists, and their unequivocal decision is required to qualify a gene to either Tier 1 or Tier 2 of the CGC.

In the latest COSMIC release (v86, August 2018), the CGC lists a total of 719 genes across the two Tiers. Of these, 554 have been associated with oncogenic and/or tumour suppressing activity, including 72 genes able either to promote or suppress oncogenesis depending on the tissue of origin, tumour stage, and various environmental factors. Some 134 genes have been found to promote cancers exclusively as fusion partners, while the precise roles of 31 Tier 2 genes still remain to be determined.

**Figure 2.**
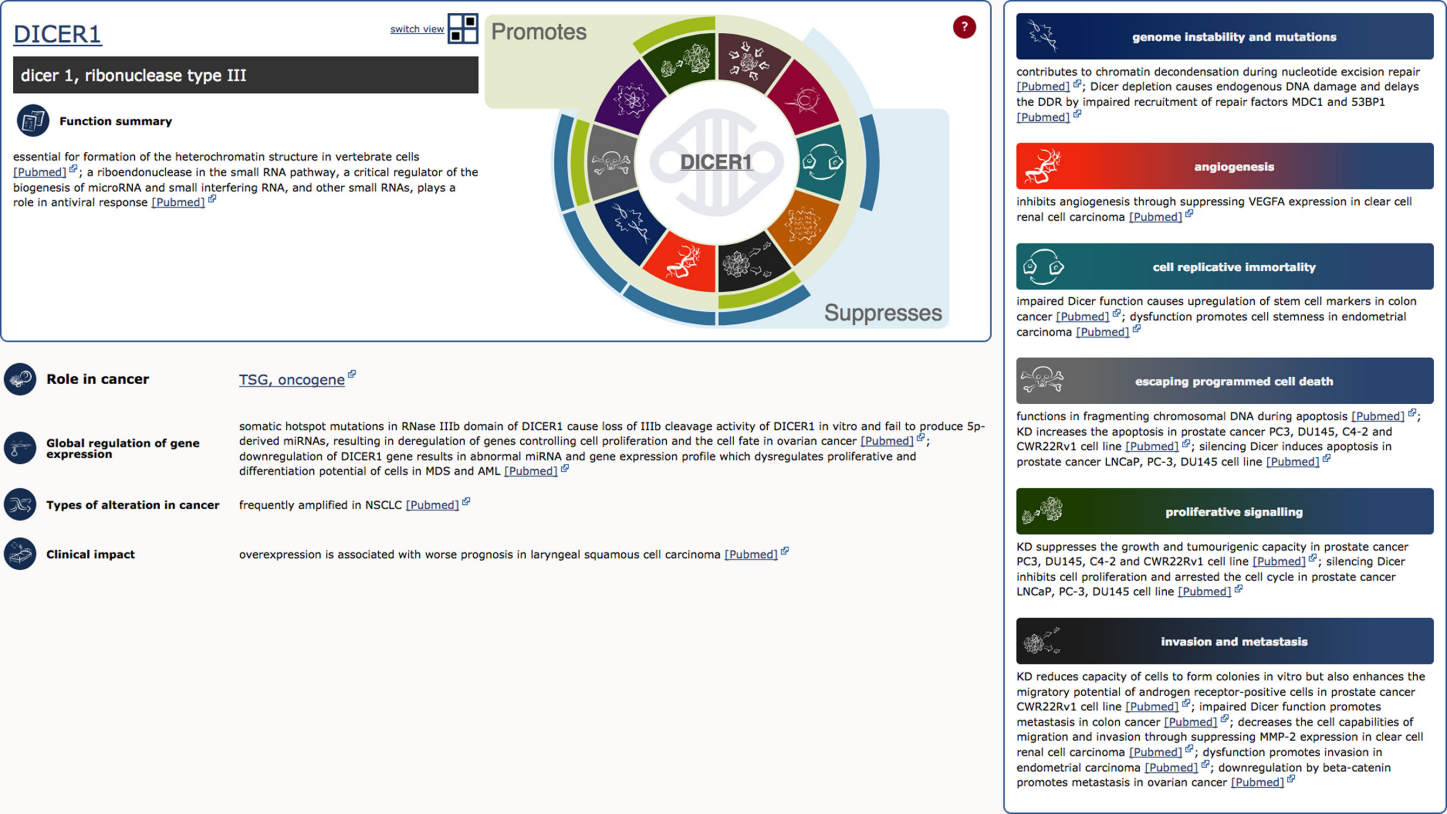
Cancer Gene Census Hallmark detail page. A gene page for DICER1 presents a spectrum of cancer-related functions of the protein coded by the gene. Involvement in each of the relevant hallmarks of cancer is concisely characterized with the indication whether the protein in its wild-type form promotes or suppresses each hallmark. All the information is referenced with links to the literature source via PubMed provided on the page.

A new section of the CGC, developed in collaboration with Open Targets (8), is focused on functional descriptions of cancer genes. Data from experimental studies are scrutinized and curated to characterize the impact of each gene on the 10 hallmarks of cancer (7). All of this information is fully referenced and presented on the new hallmark pages together with brief summaries of normal gene function and of the impact of mutations on gene dysfunction (Figure 2).

### Expert and exhaustive curation

Genes that are targeted for manual curation are generally prioritized by genes in Tier 1 of the Cancer Gene Census genes. When a new cancer gene is added to the CGC at Tier 1, an exhaustive literature search is performed in PubMed to identity any publications reporting somatic mutations in cancer. At the point at which a new gene is selected for comprehensive curation, these papers are scrutinized for mutation data as well as clinical details and phenotype information. The data are curated from both targeted gene studies and from whole genome screens, so that the full range of reported somatic mutations are represented in COSMIC. Each quarterly database release includes data for new CGC cancer genes.

Complementing the continuous addition of data for new cancer genes, COSMIC expert curators also complete a focused curation effort for each database release, centred on a particular gene or disease, which allows rapid and comprehensive coverage of new discoveries in the scientific literature. For a gene-focused curation, a literature search is performed to identify any publications with mutation data relevant to that gene that are not currently included in COSMIC. Data for the genes GNAS, GNAQ and GNA11, CTNNB1, TET2, SMAD4, VHL, PIK3CA and TERT have been updated in this way since November 2016. Since many of the publications processed as part of a gene-focus curation will also include data on additional cancer genes, these are also updated at the same time.

Similarly, focused curation is now applied to phenotypes, in order to update the somatic mutation data relating to a particular cancer and to fully represent the landscape of mutations in that cancer. Glioblastoma, the most common malignant primary tumour of the adult brain, was selected for the most recent disease focus week, with data from ∼70 new publications released in COSMIC v86 (August 2018).

Since 2016 COSMIC has included the genetics of drug resistance, annotating the novel somatic gene mutations that enable a tumour to evade therapeutic cancer drugs. These mutations are curated following an extensive review of the relevant literature, with expert curators identifying those with sufficient published evidence to be identified as resistance mutations. COSMIC v86 (August 2018) contains resistance mutation profiles across 24 drugs, detailing the recurrence of 360 unique resistance alleles across 2134 drug resistant tumours. Recent additions include MET resistance mutations in non-small cell lung cancers treated with crizotinib or capmatinib.

In addition to capturing core information on the underlying genetics of cancer, COSMIC also includes a wide variety of valuable annotations related to patients’ clinical details, their diseases and treatment. These are curated and displayed as features, attributable to an individual, tumour or sample. As many of these data points as possible are captured from a publication, although their inclusion is dependent on appropriate presentation of these data in the article.

Features that may be curated for an individual include age, either specific or a cohort, gender, and ethnicity, as well as relevant therapeutic history with respect to the screened tumour or any prior tumours. Family history, whether the individual is from a family with a syndrome such as Familial Adenomatous Polyposis, is recorded and in such cases the presence of a germline mutation in a tumour suppressor gene such as APC may be relevant and also annotated. Smoking status and alcohol intake are curated as significant environmental variables, as are exposure to radiation, UV, viruses and parasites, and chemicals/particles. Many of these data points are phenotype-specific, such as UV exposure in melanoma and human papillomavirus in cervical cancer. At the tumour level, features cover stage and grade, plus the tissue site of any reported metastases; karyotypes are recorded if especially pertinent to the tumour screened and if highlighted in the publication. Associated with curated drug resistance data, drug responses are curated as a feature, using drug-specific phrases based on RECIST (Response Evaluation Criteria in Solid Tumours) criteria (9). Clinical responses as well as in vitro responses in cell lines or xenografts are included, with particular emphasis on tyrosine kinase inhibitors. In clinical cases with multiple screened samples during tumour evolution, a sample feature indicates therapy relationships, i.e. the order of treatment lines given to an individual. Other features at the sample level highlight the exact derivation of multiple samples from a single tumour and report whether or not multiple mutations in a sample occurred on the same or different alleles.

### COSMIC-3D

**Figure 3.**
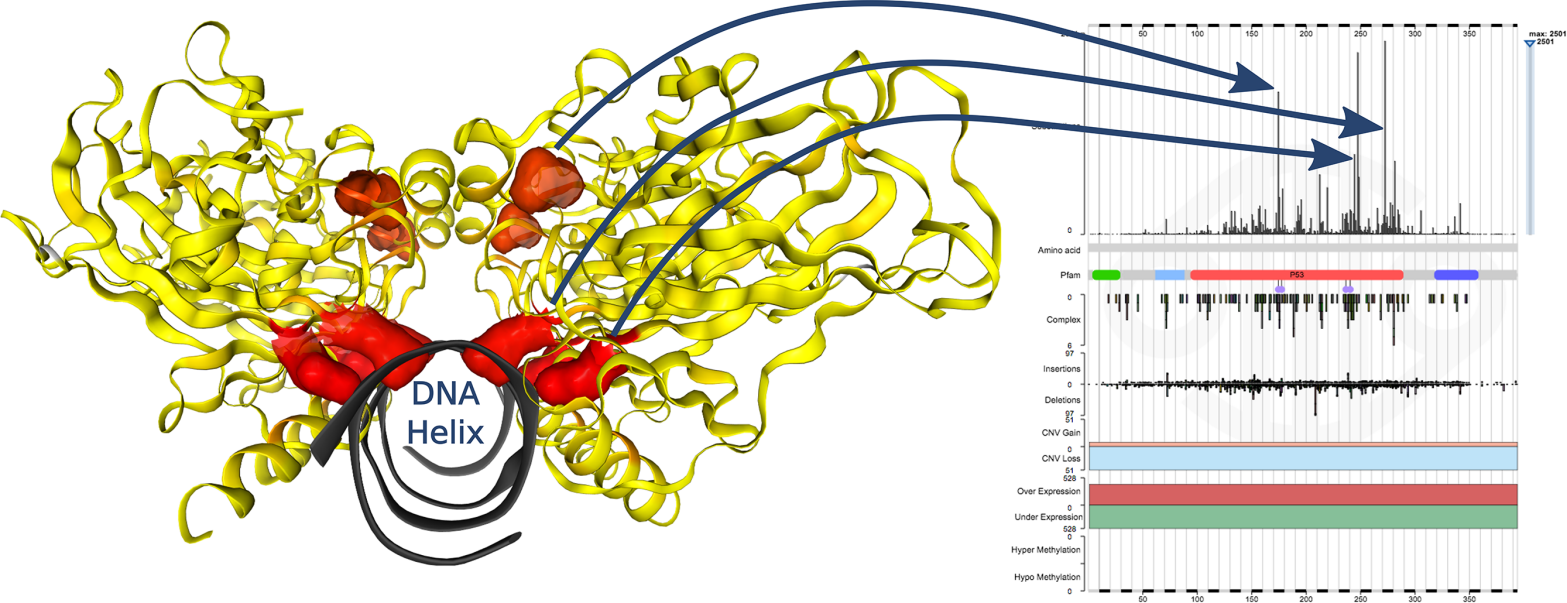
Understanding the relevance of mutation peaks in protein structure. The repertoire of mutations across TP53 are represented on a protein structure in three dimensions (PDB id: 4HJE). High-frequency substitutions are highlighted in red to show their positions relative to protein features and each other. In this example, the most frequent two mutations observed across all cancers in TP53 (at codons R248 and R273) cluster at the DNA binding surface.

Following significant expansion of COSMIC’s coverage of cancer mutation data, new tools are being added to aid the understanding of cancer genetics and drive hypothesis generation from cancer variant data. One such tool is COSMIC-3D, a platform for understanding cancer mutations in the context of three-dimensional protein structure (10) (see Figure 3). COSMIC-3D maps protein missense, in-frame deletion, and nonsense mutations to protein sequence and structure. COSMIC mutations are mapped first to UniProt (11) sequences and subsequently to wwPDB (12) protein structures via the SIFTS UniProt-to-PDB mappings (13). These data are provided through the COSMIC-3D web interface (https://cancer.sanger.ac.uk/cosmic3d), which allows interactive exploration of cancer mutation data in protein sequence and protein structural contexts, facilitating the display, understanding, and analysis of the impacts of cancer mutations. Furthermore, COSMIC-3D allows the juxtaposition of cancer mutation data with known small-molecule binding sites in the wwPDB, and with druggable binding sites as predicted using fPocket (14). By combining mutation data and known and predicted druggability, opportunities for ‘mutation guiding’ the design of small-molecules to specific cancer mutants can be explored (10).

## COSMIC AVAILABILITY

### Websites

The main COSMIC website is available at https://cancer.sanger.ac.uk/cosmic. The website is divided into two major components and several sub-sites. The primary site presents data from expert curation of the scientific literature and data from large-scale, genome-wide studies. A parallel site presents the exome sequencing and molecular profiling data from the COSMIC Cell Lines Project (https://cancer.sanger.ac.uk/cell_lines). The Cancer Gene Census site (https://cancer.sanger.ac.uk/census) provides detailed information about the 719 genes in the Census known to drive cancer, with links to the main COSMIC site and, for Tier 1 genes, to Hallmark functional descriptions. The COSMIC-3D website (https://cancer.sanger.ac.uk/cosmic3d) allows visualization of COSMIC mutation data in protein structural context.

The look-and-feel of the principle sites has recently been updated, with the aim of improving usability and navigability while maintaining the core features of each site, such as the cancer browser, genome browser and gene histogram. A consistent style has been applied, with a redesigned and better organised header and footer providing consistent links to COSMIC resources as well as tools to improve the general user experience, such as a toggle in the menu to switch between genome versions (GRCh37 versus GRCh38) and a language translation tool in the footer.

**Figure 4.**
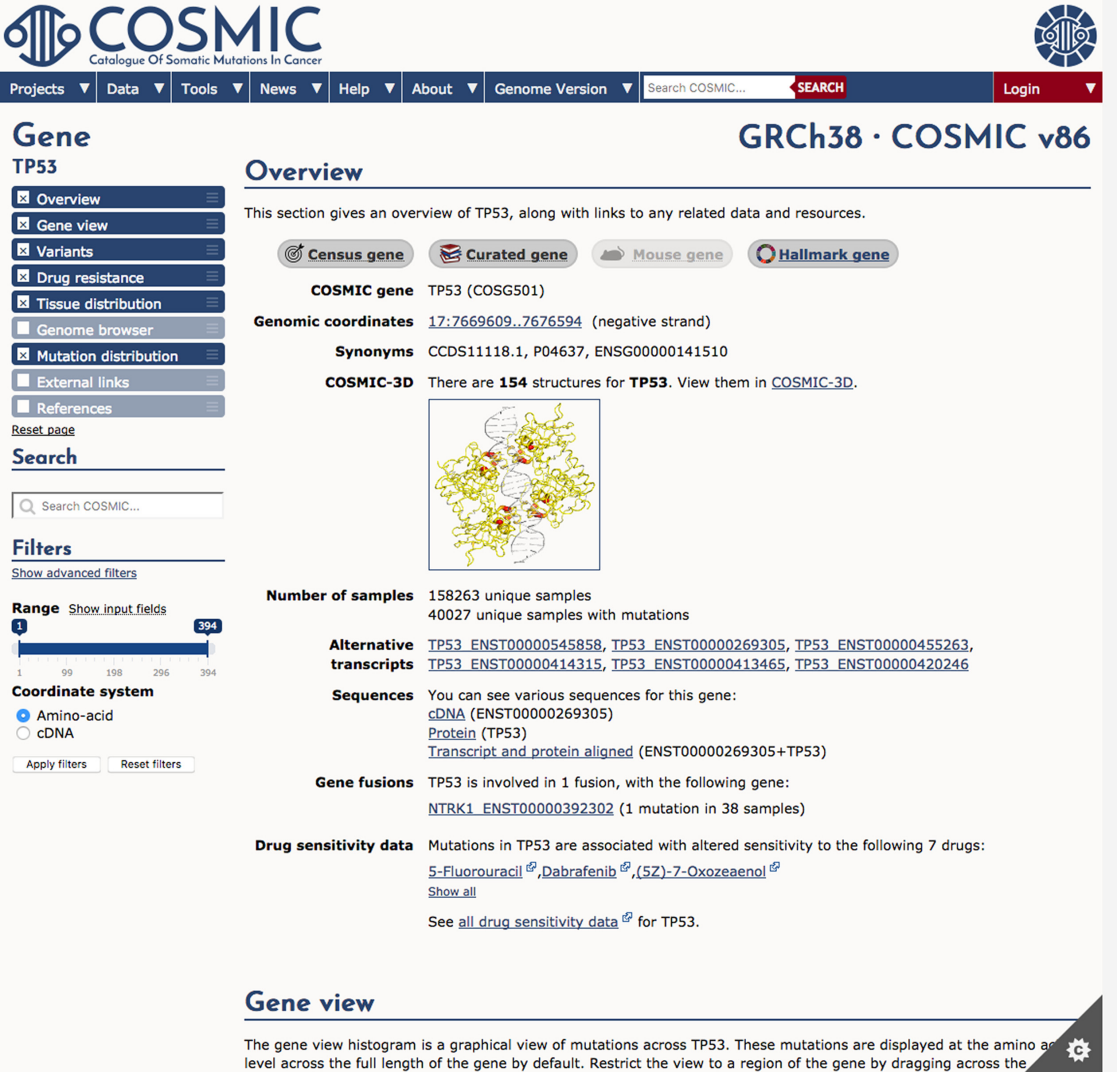
Pages showing data, such as the gene page, here shown for TP53, have been redesigned and restyled, with the page divided clearly into distinct sections.

The layout and behaviour of pages containing the majority of COSMIC data have been overhauled and improved. These data pages are organised into distinct sections, which are now displayed in a single scrollable page (rather than in a series of nested tabs as previously). Sections may be reordered within the page or hidden, allowing users to customize each different type of page and letting them bring to the fore those sections that they find most useful (see Figure 4, showing an example gene page). The order and visibility of sections within the page are recorded and used subsequently whenever a page of the same type is displayed. Filters may be applied in some areas, such as on the gene page, making it possible to narrow the focus of the data in the page by restricting the view to specific tissue types and/or histologies, particular mutation types, or to a range of bases or residues. With the overhaul of the data pages, the filter controls have been re-organised and relocated in the navigation sidebar, making them more accessible and more consistent in their placement and operation.

### Downloads

All COSMIC, Cell Lines Project, CGC and COSMIC-3D data are freely available through their respective websites. The majority of the tabular data within websites may be downloaded as comma- or tab-separated value (CSV/TSV) files from within the page itself. Since websites can inevitably answer only a subset of the questions that users may wish to ask, data are also available for bulk downloading, in the form of large, compressed CSV/TSV files or as dump files from the core Oracle database. Data from COSMIC, the Cell Lines Project and the CGC are all available for download; since COSMIC-3D represents data from COSMIC in new visual forms, no downloadable content is available. In addition to the core coding mutation content, multiple files are provided which segment information by type of variant, covering structural variants, non-coding variants, gene fusions, gene expression levels, methylation data, and resistance mutations. For the COSMIC Cell Lines Project, download files provide copy number data, average ploidy, QC data, sequence coverage statistics and genotypes.

In order to download any COSMIC data, all users must register for a COSMIC account. Academic users and those from not-for-profit organizations may download COSMIC data at no cost, but a licence fee is levied on for-profit users to support curation and infrastructure.

The primary route for downloading data is the download page on each website (https://cancer.sanger.ac.uk/cosmic/download for COSMIC and Cancer Gene Census data, or https://cancer.sanger.ac.uk/cell_lines/download for the Cell Lines Project). Once signed in, the user may download files from these pages with a single click, or, for certain files, they may choose to download only part of a file, filtered according to gene, sample or cancer type.

Prior to COSMIC release v86, data could be also downloaded en masse from a secure FTP server (SFTP), but the SFTP protocol is not well suited for use in the context of a workflow or pipeline. To better support large-scale users of COSMIC, the release of COSMIC v86 introduces a new download tool that allows users to query the list of available files programmatically and then to download them over HTTPS. Use of the SFTP server has now been deprecated. The opportunity to use HTTPS makes the download process more easily scriptable, rendering COSMIC data more readily accessible to automated pipelines. The procedure for programmatic downloading is described in full in our help pages (https://cancer.sanger.ac.uk/cosmic/help/file_download).

### Future plans

The development of all COSMIC resources is a continuous, long-term exercise. In the CGC, the information about genes is continually updated as new discoveries in cancer biology are published. Over 250 potential CGC genes are awaiting supporting evidence for inclusion, demonstrating substantial scope to expand the CGC, and to enable the re-classification of Tier 2 genes into Tier 1. In addition to increasing the coverage of functional descriptions, future development of the CGC will also focus on the context-dependent roles of genes, and how dysfunction in these genes relates across cellular pathways. The hallmark pages are being intensively developed and approximately half of Tier 1 CGC genes (as of v86, August 2018) have been annotated with functional descriptions.

Since COSMIC was first released in 2004, there have been significant changes to the type and volume of cancer mutation data uploaded to the database. Advances in genome screening technologies have been the driving force behind the large increase in cancer mutation data, as well as the availability of copy number, gene expression and methylation variation datasets. In order to accommodate these changes in COSMIC, the database model, annotation system and pipelines have been extended and adapted considerably, enhancing standardization and interoperability with other resources. There are still many challenges ahead and in order to meet these we are planning a major redevelopment of our systems over the next 2 years. The first phase, which is currently underway, is to upgrade the annotation system and data model, which will facilitate still closer interoperability with external resources such as Ensembl (15), HGNC (16) and RefSeq (17), and with the increasing number of analytical and visualization systems in development across the bioinformatics and biomedical community. The second phase will be the design and development of a new COSMIC website to ensure this ever-expanding resource is simple to explore. Engagement with our global user base will be central to this process, which will begin with a research phase to establish the key requirements of users. Any user who would like to participate in the requirements gathering and early design stages of the redevelopment is invited to contact COSMIC (cosmic@sanger.ac.uk).
